# Partial replacement of soybean meal by white lupine seeds in the diet of dairy cows

**DOI:** 10.5713/ajas.19.0457

**Published:** 2019-08-23

**Authors:** Miroslav Joch, Václav Kudrna

**Affiliations:** 1 Department of Microbiology, Nutrition and Dietetics, Czech University of Life Sciences, Prague 16500, Czech Republic; 2 Department of Nutrition and Feeding of Farm Animals, Institute of Animal Science, Prague 10400, Czech Republic

**Keywords:** Dairy Cow, Milk Production, Soybean Meal, Lupine, Methionine

## Abstract

**Objective:**

An experiment was conducted to determine the effect of partial replacement of soybean meal (SBM) by white lupine seeds (WLS) on milk yield and quality, feed efficiency and rumen fermentation of high-yielding dairy cows.

**Methods:**

Thirty multiparous cows of two breeds (20 Holstein and 10 Czech Pied cows) in early mid-lactation received three diets (treatments) in a 3×3 Latin square design with a 28-d period. The dietary treatments were as follows: CON (control total mixed ration with SBM, no WLS), WLS30 (30% of the SBM was replaced, on a dry matter basis, by WLS), and WLS50 (50% of the SBM was replaced by WLS).

**Results:**

Feed intake by the cows was not affected (p = 0.331) by the diets. Milk production decreased with increasing proportions of WLS in the diet. Cows fed WLS50 yielded approximately 1 kg/d (p<0.001) less milk than cows fed the CON diet. The proportions of milk fat (p = 0.640), protein (p = 0.507), and lactose (p = 0.709) were not altered by the diet. For milk fat, feeding with WLS50 reduced the proportion of total saturated fatty acids (p<0.001) and increased the proportion of total monounsaturated fatty acids (p<0.001), mainly through oleic acid (p<0.001). No differences were found in feed efficiency, body weight, and blood plasma metabolites between groups. Rumen ammonia-N levels tended (p = 0.087) to increase with increasing proportions of WLS in the diet, whereas no effect of diet on rumen pH was found (p = 0.558).

**Conclusion:**

We did not identify the safe range within which raw WLS can efficiently replace SBM in the diet of high-producing dairy cows. In contrast, even partial replacement of SBM by WLS favorably changed the milk fatty acid profile.

## INTRODUCTION

Europe has become heavily dependent on soybean imports [1]. In the livestock farming sector, total EU protein crop production supplies only 30% of the protein crops consumed as animal feed in the EU [2]. Although dependency on soybean is greater in monogastrics [3], conventional European dairy farms usually incorporate imported soybean meal (SBM) as a high-protein complement to the ration of dairy cows [4]. This dependency makes the livestock sector extremely vulnerable to price volatility and trade distortion. Moreover, the use of imported feeds conflicts with the increasing public concern for the traceability of supply chains, environmental protection, and food safety.

In temperate climates where soybean cultivation is not possible, white lupine seeds (WLS) may be a significant alternative to soybean in animal nutrition [5]. The high crude protein (CP) content (up to 38%) [6] makes WLS a highly suitable high-protein complement for ruminant diets. However, fast degradation of raw lupine CP in the rumen may limit its use in high-yielding dairy cows [6]. Moreover, lupins are low in methionine [7], which may further limit milk production of dairy cows [8]. A previous experiment with sheep suggested that addition of ruminally protected methionine can improve the nutritional value of lupins for ruminants [9].

Experimental results with lactating dairy cows fed raw WLS as a replacement for SBM have not been consistent. An increase [10], decrease [11], and no change [12] in milk production have all been reported after this replacement, which does not encourage farmers to use lupine seeds as a replacement for SBM. Dairy farmers must be convinced that a safe range exists in which SBM could be replaced by WLS, at least partially, without detrimental effects on feed efficiency or milk yield and quality.

The aim of the present study was to determine the effect of partial replacement (on a dry matter [DM] basis) of SBM by raw WLS on milk yield and quality, feed efficiency, and rumen fermentation in high-yielding dairy cows. We hypothesized, that under practical conditions, SBM could be partially replaced by WLS without impairing production parameters, provided that feed is supplemented with ruminally protected methionine.

## MATERIALS AND METHODS

### Animal care

The experiment was designed and performed according to European and Czech laws. The protocol was approved by the Institutional Animal Care and Use Committee of the Institute of Animal Science in Prague. The experiment was carried out at the experimental farm (Netluky, Prague) of the Institute of Animal Science, Prague, Czech Republic.

### Experimental design, animals, and diets

Thirty multiparous (parity 3±1; mean±standard deviation) cows of two breeds (20 Holstein and 10 Czech Pied cows) in early mid-lactation (83±25 d in milk; DIM), with an average body weight of 592±76 kg and average milk yield of 42±7 kg/d were used in a replicated 3×3 Latin square design with three treatments and three experimental periods. Each period lasted 28 d, with 21 d of diet adaptation and 7 d of sample collection. Cows were stratified according to breed, pretreatment milk yield, and DIM into 10 blocks (squares) of three: one cow from each block was randomly assigned to one of three treatments (diets). Diets were offered as a total mixed ration (TMR) to avoid selection of dietary components and to maintain the desired forage to concentrate ratio (approximately 67:33 on a DM basis). The SBM was partially replaced by coarsely ground (to pass through a 4 mm screen) WLS in the concentrate mixture, as follows: control (CON; concentrate mixture containing 20% SBM on a DM basis and no WLS), WLS30 (30% of the SBM was replaced, on a DM basis, by WLS), and WLS50 (50% of the SBM was replaced by WLS) (Table 1). Chemical composition of SBM and WLS is presented in Table 2.

The cows were housed in a free-stall barn with free access to water and were milked twice a day at 05:30 and 16:30. The TMR was offered *ad libitum*. Fresh TMR was prepared and delivered to the barn twice a day at approximately 04:00 and 16:00, and feeding troughs were refilled with a shovel five times per day.

### Sampling and analysis

Individual TMR intake was continuously recorded using electronic balance troughs (Insentec, B.V., Marknesse, the Netherlands). The DM intake was calculated by adjusting daily as-fed feed intake to the DM percentage of the diet.

The chemical composition of feed samples was determined according to standard methods [13–15]. The DM was determined by drying feed samples at 105°C to a constant weight. AOAC (2005) procedures were used to determine CP (954.01) and ash (942.05) content. Ether extract was analyzed according to procedure 920.39 (AOAC, 1995). The CP (6.25×N) and ether extract content were analyzed using a Kjeltec auto 1030 analyzer (Tecator AB, Höganäs, Sweden) and a Soxtec 1043 (Tecator AB, Sweden), respectively. Neutral detergent fiber, exclusive of residual ash, was assayed with heat-stable amylase [16]. Acid detergent fiber was determined according to procedure 973.18 of AOAC International (2000) and expressed exclusive of residual ash.

Individual cow milk yields were recorded daily. Milk samples were collected from each cow on two consecutive milkings (morning and evening) on days 24 and 26 of each experimental period. Samples from the morning and evening milkings were pooled proportionate to the individual milk yield of each milking and analyzed for milk fat, protein, and lactose concentrations by infrared spectroscopy (Foss FT2, MilkoScan, Foss Electric, Hillerod, Denmark).

The fatty acid (FA) composition of the milk fat was determined as described by Volek et al [17]. Briefly, FA were determined after chloroform-methanol extraction of total lipids. Methyl esters were analyzed using an HP 6890 gas chromatograph (Agilent Technologies, Inc., Santa Clara, CA, USA). One-microliter samples of solutions of FA methyl esters in hexane were injected at a 1:40 split ratio. Polyunsaturated fatty acid 1 (PUFA 1), PUFA 2, PUFA 3, and 37 Component FAME Mix (Supelco, Bellefonte, PA, USA) were used as analytical standards.

On day 23 of each experimental period, blood samples were collected from the coccygeal vein 4 h after the morning feeding, placed in heparinized tubes, immediately centrifuged at 797×*g* for 20 min [18], and aliquoted into 1-mL tubes. The tubes were stored at −18°C until analysis of plasma glucose, non-esterified fatty acids (NEFA) and blood urea nitrogen (BUN).

Rumen fluid samples (~100 mL) were collected using an oral rumen tube and a vacuum pump. The pH of the rumen fluid was measured using a pH meter (pH 700, Eutech Instruments, Singapore). The samples were immediately placed on ice and transported to the laboratory. A 1-mL aliquot was mixed with 20 μL of 9 M H_2_SO_4_ and stored at −18°C until analysis of ammonia-N. Ammonia-N concentrations were determined using the phenol-hypochlorite reaction [19]. However, the samples for analysis of total and individual volatile FA concentrations were unintentionally damaged due to technical issues and could not be used.

Body weight was measured twice a day after milking throughout the experiment using an electronic livestock scale (AfiWeigh scale, Afimilk Ltd, Kibbutz Afikim, Israel).

### Calculations

Fat-corrected milk (FCM; 4% of fat) yield was calculated according to NRC (2001) [20], as follows:

FCM (kg/d)=milk yield (kg/d)×(0.4+0.15×fat [%]).

Energy-corrected milk (ECM) yield was calculated as follows:

$$\begin{array}{l} \text{ECM }(\text{kg}/\text{d})=\text{milk yield }(\text{kg}/\text{d})\times (383\times \text{fat }[\%]+242\times \text{protien}\\ {}[\%]+165.4\times \text{lactose }[\%]+20.7)/3,140. \end{array}$$

### Statistical analyses

Data were analyzed as a replicated 3×3 Latin square design using the MIXED procedure of SAS (SAS enterprise guide version 6.1, SAS Institute Inc., Cary, NC, USA). The model was as follows:

$${\text{y}}_{ijkl}=\mu +\tau_{i}+\alpha_{j}+\beta_{k}+{\text{c}}_{l}{\left(\beta \right)}_{k}+{ɛ}_{ijkl}$$

where y*_ijkl_* is the observation of cow *l* with treatment *i* at period *j* in square *k*; μ is the overall mean; τ*_i_* is the fixed effect of treatment *i* (*i* = 1 to 3); α*_j_* is the fixed effect of period *j* (*j* = 1 to 3); β*_k_* is the fixed effect of square *k* (*k* = 1 to 10); c*_l_*(β)*_k_* is the random effect of cow *l* (*l* = 1 to 30) within the square; and ɛ*_ijkl_* is the random error. The results are reported as least squares means. Differences were considered significant at p<0.05. Trends are discussed at 0.05≤p<0.10. When a significant effect of treatment was detected, a Tukey adjustment was used to control for multiple comparisons at a probability level of p<0.05.

## RESULTS

### Feed intake, milk yield, and feed efficiency

Dry matter intake was not affected (p = 0.331) by the diet. Milk yield decreased with increasing WLS proportions in the diet (Table 3). Cows fed the WLS50 diet yielded approximately 1 kg/d less milk than cows fed the CON diet. Similarly, FCM and ECM yields were higher (p<0.001) in animals on the CON diet than in those on the WLS diets. Milk fat, protein, and lactose were not influenced by the dietary protein source. Feed efficiency, expressed as kg of milk/kg of DM intake, was not affected (p = 0.110) by the treatment. Replacement of SBM by WLS had no effect on nitrogen use efficiency (NUE; g milk N/100 g N intake; p = 0.822).

### Fatty acid profile of milk fat

The diet-dependent FA profile of the milk fat is presented in Table 4. The replacement of SBM by WLS (WLS30 and WLS50) increased the arachidic acid (p = 0.002) and docosapentaenoic acid (DPA; p = 0.004) concentrations and decreased the concentrations of palmitoleic acid (p<0.001) and conjugated linoleic acid (CLA; isomer t10,c12; p = 0.005) compared to the CON diet. In addition, the WLS50 diet increased the concentrations of oleic acid (p<0.001) and total monounsaturated fatty acids (MUFA, p<0.001) and decreased the concentrations of lauric acid (p = 0.005) and total saturated fatty acid (SFA, p<0.001). The concentrations of caproic and stearic acids tended to increase with the WLS diets compared to the CON diet.

### Rumen fermentation parameters and blood plasma metabolites

Ammonia-N concentrations in rumen fluid tended (p = 0.087) to increase with increasing proportions of lupine seeds in the diet (Table 3). Rumen pH was unaffected by the treatments (p = 0.558).

Plasma concentrations of glucose, NEFA (p = 0.512), and BUN (p = 0.414) remained unaffected by the type of diet (Table 3).

### Mean body weight and body weight change

Inclusion of WLS in the diet did not affect the mean body weight (p = 0.350) or body weight change (p = 0.437) of the dairy cows (Table 3).

## DISCUSSION

The high degradability of CP in rumen and low content of some essential amino acids, notably methionine, are two main factors that lower the value of WLS as a protein supplement in the diet of high-yielding dairy cows [7,21]. According to tables of the French National Institute for Agricultural Research (INRA), the effective degradability of white lupin CP is markedly higher compared to SBM (86% vs 63% for a passage rate of 6%/h) [22]. Therefore, complete replacement of CP from SBM an equal amount of CP from WLS could result in ammonia production in excess of bacterial need, leading to reduced efficiency of use of dietary N [20]. The protein value of lupine seeds can be increased by heat treatment [5], although evidence does not support the use of heating as a cost-effective measure for practical diets [21]. Furthermore, the protein value of lupine for milk production can be improved by the addition of ruminally protected methionine, since WLS protein is deficient in methionine relative to needs for milk protein synthesis in dairy cows [21]. Consequently, we hypothesized that, in high-yielding dairy cows, a diet supplemented with ruminally protected methionine SBM could be partially replaced by WLS without negative effects on cow performance.

Our hypothesis could not be confirmed by our results. The cows fed WLS30 and WLS50 diets, where 0.50 and 0.85 kg/cow per day (on DM basis) of SBM, respectively, was replaced by WLS, produced less milk than cows fed the CON diet without WLS. The effect was more pronounced as the proportion of WLS in the diet increased. Similar results were obtained for FCM and ECM, likely due to both lower milk production and slightly smaller proportion of milk fat observed for cows fed with WLS diets. This confirms previous findings by Guillaume et al [11], who reported that lactating cows fed 2.6 kg/d of lupine (on DM basis) produced less milk than cows fed 2 kg/d of SBM. There are several possible explanations for the reduced milk production observed with WLS supplementation.

The decreased milk yield reported in the present study may be attributed to a combination of lower CP content in the WLS diets and an intake of DM that was below the requirement. The lower CP content in the WLS diets was intentional and in accordance with the current trend of reducing CP in the diet of dairy cows to increase N use efficiency and reduce N pollution [23]. The CP in the diets decreased as the proportion of WLS increased (Table 1) because WLS contained only 59.2% of the CP content found in SBM (Table 2). However, the difference in CP content between diets was up to 0.7% of CP/kg of DM. Although the diets were not isonitrogenous, they were formulated to meet or exceed protein requirements [20]. However, because the cows in all three diet groups ate less than expected, the lower CP content in the WLS diets could elicit a lower milk yield compared to the CON diet.

The energy content was almost identical among the experimental diets (Table 1). Thus, the recorded decrease in milk production was not likely to have been due to energy unavailability in the WLS diets. Nevertheless, the energy status of cows fed the CON diet might have improved due to more stable degradation of CP in SBM, which may have stimulated rumen microbes to extract more energy from feedstuff in the rumen [5].

Additionally, the reduced milk yield associated with the WLS diets may be connected with reduced amino acids flow into the duodenum due to the extensive ruminal degradation of lupine CP [24]. In our study, a more intense degradation of dietary CP in cows fed WLS diets was indicated by higher levels of rumen ammonia-N, which tended to increase with increasing WLS proportions. In keeping with this, the higher CP degradation rate of lupine seeds compared to SBM has been previously reported [6,25]. Overall, however, our other results did not suggest that the efficiency of use of dietary CP was impaired with WLS supplementation. The NUE was above average [26] in all three experimental diets, likely because of relatively low CP intake. In addition, there was no difference in BUN between diets.

Replacement of SBM by WLS had no effect on the concentrations of milk components, which is in agreement with the findings of May et al [10]. Other authors found that a diet supplemented with raw WLS increased the concentration of milk fat [12] or decreased the concentration of milk protein [5,11]. This discrepancy could be explained by the different levels of replacement of SBM by WLS. In our study, supplementation with WLS was considerably lower compared to the above-mentioned studies. In addition, supplementation with ruminally protected methionine could prevent the decrease in milk protein associated with WLS diets because methionine, together with lysine, may be limiting for milk protein synthesis with a lupine diet [8,21].

In this study, even small-scale replacement of SBM by WLS altered the concentrations of several individual FA. This could be explained by the different FA profiles of these two protein supplements [12] and the more than fivefold greater ether extract content in WLS compared to SBM (Table 2). As expected, differences were more pronounced in cows fed a greater proportion of WLS. Feeding with WLS50 decreased the levels of medium-chain SFA, primarily capric (C10:0), lauric (C12:0), and myristic (C14:0) acids. This might be related to the higher proportion of fat in the WLS50 diet since the *de novo* synthesis of these FA declines with fat intake [27]. In contrast, two long-chain SFA, stearic (C18:0) and arachidic (C20:0) acids, tended to increase or increase, respectively. The increase in stearic acid levels could be due to rumen hydrogenation of oleic acid (C18:1), the main FA in WLS fat [21].

Regarding MUFA, the increased concentration of oleic acid (C18:1 n9) in the milk is likely evidence of a higher transfer of this FA to milk from the WLS diet. Although the mechanism by which the palmitoleic acid (C16:1 n7) concentration was reduced with the WLS diets is not clear, this reduction is in agreement with previous findings [12].

Among the PUFA, the levels of two minor FA, arachidonic acid (C20:4 n6) and DPA (C22:5 n3), were increased by the WLS diets, whereas two isomers of CLA (C18:2 c9,t11 and C18:2 t10,c12) were decreased by the WLS diets compared to the CON diet. Diet can have a major impact on milk fat content, but substantial variation in milk fat CLA content (C18:2 c9,t11) is observed among individuals consuming the same diet [28]. The major source of CLA (C18:2 c9,t11) in milk fat is endogenous synthesis from vaccenic acid (C18:1 n7t) via Δ^9^-desaturase [29]. Therefore, CLA levels may have decreased due either to the lower activity of Δ^9^-desaturase or lower availability of vaccenic acid (C18:1 n7t). The latter is indicated by the reduced concentration of vaccenic acid (C18:1 n7t) in milk fat.

With WLS inclusion, although the magnitude of change in the FA profile was small, the direction of changes was a relatively clear. The concentrations of SFA were reduced and those of MUFA increased. These changes suggest that replacing SBM by WLS could result in the production of milk with a FA profile beneficial for human health [30].

The blood parameters measured in the present study were within the reference ranges for dairy cows [31,32]. The results of plasma glucose, NEFA, and BUN suggest an unchanged nutritional status in the dairy cows. The low levels of NEFA indicate limited lipomobilization and a positive energy balance, which is in accordance with the weight gain data (Table 3).

## CONCLUSION

Under the conditions of the present study, small-scale replacement of SBM by raw WLS compromised milk production in high-yielding dairy cows. This could be related to a less effective use of degradable protein in rumen and/or lower supply of rumen undegradable protein in cows fed WLS diets. The results did not confirm our hypothesis that in the diets supplemented with ruminally protected methionine SBM could be partially replaced (on a DM basis) by WLS without adverse effect on performance of dairy cows. In contrast, even partial replacement of SBM by WLS elicited favorable changes in the FA profile of milk.

## Figures and Tables

**Table 1:** Ingredients and chemical composition of the experimental diets (% DM unless otherwise stated)

Items	Diet1)		

	CON	WLS30	WLS50
Ingredient (% DM)			
Forage	67.3	67.3	67.3
Corn silage	26.9	26.9	26.9
Alfalfa silage	16.6	16.6	16.6
Ensiled crushed corn cobs with bracts	12.0	12.0	12.0
Brewers grain	6.4	6.4	6.4
Alfalfa hay	5.4	5.4	5.4
Concentrate	32.7	32.7	32.7
Wheat	6.7	6.7	6.7
Barley	6.7	6.7	6.7
Rapeseed meal	5.8	5.8	5.8
Soybean meal	6.4	4.5	3.2
White lupine seeds	-	1.9	3.2
SoyPass2)	3.2	3.2	3.2
LactoPlus3)	1.6	1.6	1.6
Vitamin and mineral mix4)	1.3	1.3	1.3
Sodium bicarbonate	0.3	0.3	0.3
Methipass S5)	0.7	0.7	0.7
Chemical composition (% DM)			
Dry matter (% as fed)	47.6	47.6	47.6
Crude protein	19.2	18.8	18.5
Ether extract	4.7	4.9	5.0
Ash	5.8	5.7	5.7
ADF	20.4	20.7	20.9
NDF	41.7	41.9	42.0
Gross energy (MJ/kg DM)	19.44	19.42	19.41

DM, dry matter; ADF, acid detergent fiber; NDF, neutral detergent fiber.

1) CON, control, soybean meal; WLS30, 30% of the soybean meal replaced by white lupine seeds; WLS50, 50% of the soybean meal replaced by white lupine seeds.

2) SoyPass, a rumen bypass lignosulfonate-treated soybean meal (NOACK, Prague, Czech Republic).

3) LactoPlus, a rumen bypass fat containing at least 75% palmitic acid (Premium Vegetable Oils Sdn. Bhd., Kuala Lumpur, Malaysia)

4) Vitamin and mineral mix containing (per kg): 403,100 IU vitamin A, 73,494 IU vitamin D_3_, 1,200 mg vitamin E, 133 g Ca, 33 g P, 52 g Na, 40 g Mg, 630 mg Cu, 4,855 mg Mn, 3,160 mg Zn, 18 mg Se, 53 mg I, and 21 mg Co.

5) Methipass S (Adisseo France SAS, Antony, France) containing 10% of a rumen-protected form of methionine was added directly into the horizontal mixing wagon due to the susceptibility of the methionine protective coating to mechanical disturbance.

**Table 2:** Chemical composition of soybean meal and white lupine seeds (% DM unless otherwise stated)

Chemical composition (% DM)	Soybean meal	White lupine seeds
Dry matter (% as fed)	87.4	88.2
Crude protein	49.6	29.4
Ether extract	2.2	11.9
Ash	7.7	4.8
ADF	7.2	22.0
NDF	15.9	28.7
RUP1)	17.4	5.6
RDP2)	32.3	23.8
Gross energy (MJ/kg DM)	20.45	19.81

DM, dry matter; ADF, acid detergent fiber; NDF, neutral detergent fiber; RUP, rumen undegradable protein; RDP, rumen degradable protein.

1) RUP, calculated according to INRA [22].

2) RDP, calculated according to INRA [22].

**Table 3:** Feed intake, milk production and composition, blood plasma metabolites, rumen fermentation parameters, and body weight of cows fed the control diet or diets containing white lupine seeds

Items	Treatment1)			SEM	p-value

	CON	WLS30	WLS50		
Intake					
Dry matter (kg/d)	20.9	20.6	21.1	0.22	0.331
Milk					
Yield (kg/d)	36.1^a^	35.7^ab^	35.2^b^	0.25	<0.001
FCM2) yield (kg/d)	35.5^a^	34.6^b^	34.4^b^	0.26	<0.001
ECM3) yield (kg/d)	35.5^a^	34.7^b^	34.4^b^	0.25	<0.001
Fat (%)	3.89	3.81	3.84	0.041	0.640
Protein (%)	3.22	3.23	3.21	0.021	0.507
Lactose (%)	4.86	4.86	4.85	0.011	0.709
Feed efficiency					
kg of milk/kg of DMI	1.83	1.85	1.71	0.034	0.110
NUE (g milk N/100 g N intake)	30.1	31.1	31.4	1.15	0.822
Blood					
Glucose (mmol/L)	3.15	3.24	3.20	0.029	0.227
NEFA (mmol/L)	0.11	0.10	0.11	0.003	0.512
BUN (mg/dL)	20.0	20.5	20.7	0.34	0.414
Rumen fluid					
Ammonia-N (mg/dL)	12.19	13.16	13.59	0.370	0.087
pH	6.80	6.72	6.74	0.047	0.558
Body weight					
Mean (kg)	609	611	610	3.1	0.350
Change (kg/d)	0.47	0.43	0.30	0.065	0.437

SEM, standard error of the mean; FCM, fat-corrected milk; ECM, energy-corrected milk; DMI, dry matter intake; NUE, nitrogen use efficiency; NEFA, non-esterified fatty acids; BUN, blood urea nitrogen.

1) CON, control, soybean meal; WLS30, 30% of the soybean meal replaced by white lupine seeds; WLS50, 50% of the soybean meal replaced by white lupine seeds.

2) Fat-corrected milk (containing 4% fat) yield calculated as follows: FCM (kg/d) = milk yield (kg/d)×[0.4+0.15×fat (%)].

3) Energy-corrected milk yield calculated as follows: ECM (kg/d) = milk yield (kg/d)×[383×fat (%)+242×protein (%)+165.4×lactose (%)+20.7]/3140.

a,b Means in the same row with different superscripts differ (p<0.05).

**Table 4:** Fatty acid profile (g/100 g total fatty acids) in the milk of cows fed the control diet or diets containing white lupine seeds

Fatty acid	Treatment1)			SEM	p-value

	CON	WLS30	WLS50		
SFA					
Butyric (C4:0)	2.64	2.62	2.59	0.020	0.343
Caproic (C6:0)	1.75	1.83	1.81	0.016	0.060
Caprylic (C8:0)	1.17	1.18	1.16	0.013	0.452
Capric (C10:0)	2.87^ab^	2.97^a^	2.82^b^	0.033	0.016
Lauric (C12:0)	3.44^a^	3.46^a^	3.29^b^	0.041	0.005
Myristic (C14:0)	11.3^ab^	11.4^a^	11.1^b^	0.059	0.022
Palmitic (C16:0)	36.1	36.1	35.8	0.14	0.291
Stearic (C18:0)	9.9	10.2	10.1	0.08	0.084
Arachidic (C20:0)	0.15^b^	0.16^a^	0.16^a^	0.003	0.002
Other SFA	2.16	2.09	2.11	0.017	0.090
Total SFA	61.6^a^	61.7^a^	60.8^b^	0.16	<0.001
MUFA					
Myristoleic (C14:1 n5)	1.03	1.04	1.01	0.016	0.508
Palmitoleic (C16:1 n7)	1.54^a^	1.45^b^	1.40^b^	0.022	<0.001
Vaccenic (C18:1 n7t)	0.73^a^	0.68^b^	0.74^a^	0.012	<0.001
Oleic (C18:1 n9)	19.7^b^	19.5^b^	20.4^a^	0.13	<0.001
Elaidic (C18:1 n9t)	0.40	0.38	0.41	0.008	0.325
Other MUFA	1.06^a^	0.98^b^	1.04^ab^	0.014	0.012
Total MUFA	24.5^b^	24.1^b^	25.0^a^	0.134	<0.001
PUFA					
CLA (C18:2 c9,t11)	0.49^a^	0.45^b^	0.46^ab^	0.009	0.006
CLA (C18:2 t10,c12)	0.012^a^	0.009^b^	0.009^b^	0.0004	0.005
Linoleic (C18:2 n6)	2.11	2.08	2.13	0.023	0.644
α-Linolenic (C18:3 n3)	0.40	0.39	0.41	0.004	0.118
γ-Linolenic (C18:3 n6)	0.03	0.03	0.03	0.001	0.473
Arachidonic (C20:4 n6)	0.166^b^	0.177^a^	0.174^ab^	0.0022	0.020
EPA (C20:5 n3)	0.037	0.038	0.038	0.0011	0.967
DPA (C22:5 n3)	0.059^b^	0.068^a^	0.067^a^	0.0015	0.004
DHA (C22:6 n3)	0.009	0.009	0.011	0.0004	0.129
Other PUFA	0.75	0.75	0.78	0.008	0.118
Total PUFA	4.06	4.00	4.10	0.036	0.277
PUFA n6/PUFA n3	5.36	5.40	5.25	0.053	0.392

SEM, standard error of the mean; SFA, saturated fatty acids; MUFA, monounsaturated fatty acids; PUFA, polyunsaturated fatty acids; CLA, conjugated linoleic acids; EPA, eicosapentaenic acid; DPA, docosapentaenoic acid; DHA, docosahexaenic acid.

1) CON, control, soybean meal; WLS30, 30% of the soybean meal replaced by white lupine seeds; WLS50, 50% of the soybean meal replaced by white lupine seeds.

a,b Means in the same row with different superscripts differ (p<0.05).
